# Pharmacokinetics and safety of two Voriconazole formulations after intravenous infusion in two doses in healthy Chinese subjects

**DOI:** 10.1186/s40360-023-00652-3

**Published:** 2023-03-03

**Authors:** Xin Li, Chenjing Wang, Ping Shi, Yanping Liu, Ye Tao, Pingping Lin, Ting Li, Haixun Hu, Feifei Sun, Shuqin Liu, Yao Fu, Yu Cao

**Affiliations:** 1 Phase I Clinical Research Center, The Affiliated Hospital of Qingdao University, Qingdao, 266003 China; 2 Clinical Research Department, Qilu Pharmaceutical Co., Ltd, Jinan, 250108 China

**Keywords:** Voriconazole, Pharmacokinetics, Bioequivalence, Safety

## Abstract

**Background:**

Voriconazole is a second-generation triazole that is used to prevent and treat invasive fungal infections. The purpose of this study was to evaluate the pharmacokinetic equivalency of a test formulation and reference formulation (Vfend®) of Voriconazole.

**Materials and methods:**

This was a randomized, open-label, single-dose, two-treatment, two-sequence, two-cycle, crossover phase I trial. The 48 subjects were equally divided into 4 mg/kg and 6 mg/kg groups. Within each group, the subjects were randomized 1:1 to the test or reference formulation.. After a 7-day washout period, crossover formulations were administered. The blood samples were collected at 0.5, 1.0, 1.33,1.42,1.5, 1.75, 2.0, 2.5, 3.0, 4.0, 6.0, 8.0, 12.0, 24.0, 36.0, 48.0 h later in the 4 mg/kg group, while at 0.5, 1.0, 1.5, 1.75, 2.0, 2.08, 2.17, 2.33, 2.5, 3.0, 4.0, 6.0, 8.0, 12.0, 24.0, 36.0, 48.0 h later in the 6 mg/kg group. The plasma concentrations of Voriconazole were determined by Liquid chromatography-tandem mass spectrometry (LC–MS/MS). The safety of the drug was evaluated.

**Results:**

The 90% confidence intervals (CIs) of the ratio of geometric means (GMRs) of C_max_, AUC_0-t_, and AUC_0-∞_ in both 4 mg/kg and 6 mg/kg groups were within the prespecified bioequivalence limits between 80 ~ 125%. In the 4 mg/kg groups, 24 subjects were enrolled and completed the study. The mean C_max_ was (2.552 ± 0.448) μg/mL, AUC_0-t_ was (11.875 ± 7.157) h*μg/mL and AUC_0-∞_ was (12.835 ± 9.813) h*μg/mL after a single dose of 4 mg/kg test formulation. The mean C_max_ was (2.615 ± 0.464) μg/mL, AUC_0-t_ was (12.500 ± 7.257) h*μg/mL and AUC_0-∞_ was (13.416 ± 9.485) h*μg/mL after a single dose of 4 mg/kg reference formulation. In the 6 mg/kg groups, 24 subjects were enrolled and completed the study. The mean C_max_ was (3.538 ± 0.691) μg/mL, AUC_0-t_ was (24.976 ± 12.364) h*μg/mL and AUC_0-∞_ was (26.212 ± 14.057) h*μg/mL after a single dose of 6 mg/kg test formulation. The mean C_max_ was (3.504 ± 0.667) μg/mL AUC_0-t_ was (24.990 ± 12.455) h*μg/mL and AUC_0-∞_ was (26.160 ± 13.996) h*μg/mL after a single dose of 6 mg/kg reference formulation. Serious adverse event (SAE) was not observed.

**Conclusion:**

In both 4 mg/kg group and 6 mg/kg group, equivalent pharmacokinetic characteristics that satisfied the criteria of bioequivalence for both test and reference formulations of Voriconazole.

**Trial registration:**

NCT05330000 (15/04/2022).

**Supplementary Information:**

The online version contains supplementary material available at 10.1186/s40360-023-00652-3.

## Introduction

Voriconazole is a second-generation triazole antifungal drug synthesized artificially. Although its structure is similar to that of fluconazole, its antibacterial efficacy is improved [1]. It plays an antifungal role by inhibiting cytochrome P450 dependent enzymes, interfering with ergosterol synthesis, inhibiting cell membrane synthesis. The antibacterial spectrum of Voriconazole includes Aspergillus, Fusarium and Scedosporium, as well as Candida in yeast [2]. Currently, the varieties sold on the market include 50 mg and 200 mg oral tablets, 40 mg / mL oral suspension and 200 mg injection.

This is a randomized, open, two sequence, two cycle, crossover clinical study. The main purpose of the study was to evaluate the bioequivalence between experimental formulation and the reference formulation (Vfend ®) at 4 mg/kg and 6 mg/kg doses. The secondary purpose of the study was to compare the safety of the two formulations.

## Methods

### Ethics

The trial was performed abiding by the Declaration of Helsinki [3], Good Clinical Practice (GCP) [4] and the guidelines of China National Medical Products Administration (NMPA). The protocol, informed consent and other relevant documents were independently approved by the Medical Ethics Committee of the Affiliated Hospital of Qingdao University and were numbered QYFYEC 2020–018-01. Any deviation or violation of the protocol during the trial overshoot has been reported to the ethics committee.

### Subjects

Volunteers who meet the following conditions can be included in the trial: 1) healthy men and women aged 18–45, with a body mass index of 19-28 kg / m^2^ (including the boundary value). The weight of male is not less than 50.0 kg, and that of female is not less than 45.0 kg. 2) Good health, no history of cardiovascular system, digestive system, urinary system, endocrine system, blood system, respiratory system, nervous system, etc. The following examination results are normal or abnormal with no clinical significance. The examinations include: body temperature, blood pressure, heart rate, hematology, blood biochemistry, urinalysis, serological detection of hepatitis B virus, hepatitis C virus, human immunodeficiency virus (HIV) and Treponema pallidum, coagulation, 12 lead electrocardiogram (ECG), alcohol breath test, drug screening and female pregnancy test. 3) The subjects and their spouses did not have family planning within 6 months and chose appropriate contraceptive methods. 4) Before the study, all subjects were informed of the purpose, process, benefits and risks of the study and voluntarily signed informed consent.

Subjects who were allergic to study drugs, smoke, drink alcohol, and participate in other clinical trials within 3 months could not participate in the trial.

### Study design

This is an open, randomized, single dose, two cycle, crossover design trial. In this study, 0.2 g was selected for the comparative study of safety and pharmacokinetics under the dose of 4 mg/kg and 6 mg/kg respectively. According to the technical guidelines for clinical pharmacokinetic studies of chemical drugs (2005 Edition), the number of subjects for pharmacokinetic studies is generally 8–12 in each dose group. It is suggested in the clinical trial notice that the pharmacokinetic test can refer to the requirements of bioequivalence test. The drug registration management measures mentioned that the bioequivalence test generally involves 18–24 subjects. In addition, in consideration of the possibility of subject dropouts during the trial, 24 subjects were enrolled in the low-dose group and the high-dose group respectively. The order in which each subject received the test preparation or the reference preparation would be determined by the randomization table (Table 1). The randomization table was generated by the statistical unit using the method of block randomization in SAS 9.4.

**Table 1:** Study design for the bioequivalence evaluation of Voriconazole

Groups	Number of cases	The first period	The second period
4 mg/kg group	12	T^a^	R^b^
	12	R	T
6 mg/kg group	12	T	R
	12	R	T

^a^T: test formulation, Voriconazole injection, 200 mg/vial

^b^R: reference formulation, Vfend®, 200 mg/vial

Voriconazole at a single dose of 4 mg/kg was injected with infusion pump within 80 min. 3 mL blood samples were taken from the contralateral forearm before administration and at 0.5, 1.0, 1.33,1.42,1.5, 1.75, 2.0, 2.5, 3.0, 4.0, 6.0, 8.0, 12.0, 24.0, 36.0, 48.0 h after administration. Voriconazole at a single dose of 6 mg/kg was injected with infusion pump within 120 min. 3 mL blood samples were taken from the contralateral upper arm before administration and at 0.5, 1.0, 1.5, 1.75, 2.0, 2.08, 2.17, 2.33, 2.5, 3.0, 4.0, 6.0, 8.0, 12.0, 24.0, 36.0, 48.0 h after administration. The samples were centrifuged at 2,000 g for 10 min at 4 °C to separate the plasma. The plasma samples were divided into two aliquots by disposable pipette and stored at -80 °C until bioanalysis. The half-life of single intravenous drip of 6 mg/kg is about 6.4 h. Since the washout period should be more than 7 times the half-life, so the interval between two administration was set as 7 days. The operation of the two periods was consistent.

### Safety assessment

The safety was assessed by monitoring vital signs and laboratory tests. Vital signs, such as body temperature, blood pressure, and heart rate, were measured before administration and at 0.5, 1.33, 3.0, 6.0, 12.0, 24.0, 48.0 h after administration in 4 mg/kg group, while at 0.5, 2.0, 4.0, 8.0, 12.0, 24.0, 48.0 h after administration in 6 mg/kg group. Before removal from this study, the subjects would receive physical examination, vital signs assessment (blood pressure, pulse, body temperature), electrocardiogram examination, laboratory examination, including hematology, urinalysis, blood biochemistry, coagulation function, female blood pregnancy. All adverse events onset within 7 half-life of the drug were followed up until they returned to normal or stable. Adverse events (AEs) were recorded, including clinical symptoms and signs, severity, occurrence and end time, duration, treatment measures, and causal relationship to the study drug.

### Bioanalysis

Voriconazole were determined by liquid chromatography tandem mass spectrometry (LC–MS / MS) by Shanghai Fangda Biotechnology Co., Ltd. The equipment used in the determination process was acquity ultra-high performance liquid chromatography (Nexera UHPLC LC-30A, Shimadzu, Japan) and mass spectrometer (SCIEX API 4000, Applied Biosystems, Canada). Under the multi reaction monitoring and positive ionization mode, the samples were pretreated with acetonitrile by protein precipitation method, and then 5 μL pretreated samples were injected into LC–MS/MS system. Analyst 1.6.3 software (Applied Biosystems, Foster City, California, USA) was used for data collection and analysis. The sample analyst always keeps blind to the random group during the test.

The method is verified fully by selectivity, accuracy, precision, calibration curve and stability. The drug concentration was linear within the range of 0.010 ~ 10.000 μg/mL. The lower limit of quantification was 0.010 μg/mL. Calibration curves were linear with the Pearson correlation coefficients ranged from 0.9950 to 0.9969. There was no significant interference in selectivity and stability.

### Pharmacokinetic analysis

All 48 subjects completed the study, and the data were included in the pharmacokinetic analysis. According to the actual blood collection time, the pharmacokinetic parameters were calculated using the non atrioventricular model by WinNonlin®8.3.1 (pharsight, St. Louis, Mo, USA). In the concentration data list, for the samples which concentration is lower than the lower limit of quantification, the samples before reaching C_max_ were calculated as zero value, and the samples after reaching C_max_ were calculated as non-quantifiable (ND). The primary pharmacokinetic (PK) parameters include the maximum plasma concentration (C_max_), the area under the plasma concentration–time curve from 0 to the last measured time point (AUC_0-t_), and the area under the plasma concentration–time curve from 0 to infinity (AUC_0-∞_). The secondary PK parameters include the observed time to C_max_ (T_max_) and the apparent terminal half-life (T_1/2_).

### Statistical analysis

Statistical analyses were performed by SAS 9.4 (SAS Institute Inc. Cary, NC, USA). The effects from subject, treatment, period, and preparation were assessed by Analysis of variance (ANOVA) performed on the logarithmically transformed C_max_, AUC_0-t_, and AUC_0-∞_. The GMRs of the primary PK parameters and the 90% confidence intervals (CIs) were calculated. If the 90% CIs for the GMRs is within the equivalent range (80 ~ 125%), it is determined that the two formulations are bioequivalent. Statistical data were presented as mean ± standard deviation (SD). The probability value less than 0.05 is considered statistically significant.

## Results

### Characteristics of the subjects

All subjects completed the study. A total of 24 subjects including 3 women and 21 men enrolled in the 4 mg/kg group. Allof them were Han nationality. Parameter, mean ± SD (range): age, 30.50 ± 7.12 years (20.00 ~ 42.00 years); weight, 66.71 ± 7.61 kg (52.50 ~ 83.50 kg); height, 169.79 ± 5.27 cm (159.00 ~ 178.00 cm); body mass index (BMI), 23.15 ± 2.39 kg × m^−2^ (19.20 ~ 26.60 kg × m^−2^).

A total of 24 subjects including 5 women and 19 men enrolled in the 6 mg/kg group. One subject is Manchu, and the other 23 cases are Han nationality. Parameter, mean ± SD (range): age, 27.00 ± 6.76 years (19.00 ~ 44.00 years); weight, 67.73 ± 9.71 kg (49.50 ~ 86.00 kg); height, 170.52 ± 7.64 cm (153.00 ~ 184.50 cm); BMI, 23.23 ± 2.44 kg × m^−2^ (19.40 ~ 27.00 kg × m^−2^).

### Pharmacokinetics

**Fig. 1:**
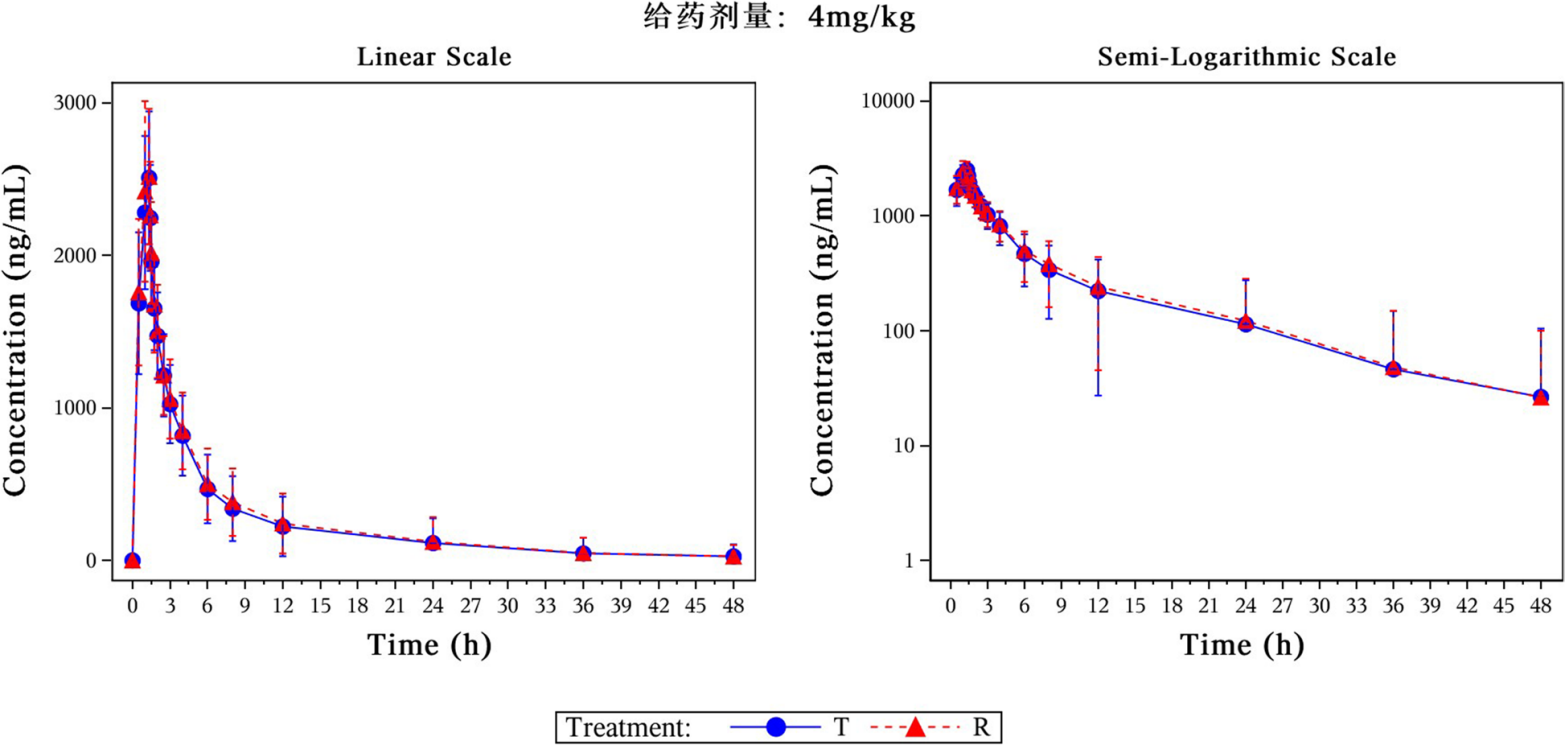
The mean plasma concentration–time curves following a single dose of test formulations or reference formulations in the 4 mg/kg group

Following a single dose of test formulations or reference formulations, the mean plasma concentration–time curves were shown in Figs. 1 (the 4 mg/kg group) and 2 (the 6 mg/kg group). Individual plasma concentration–time data of Voriconazole was show supplementary file.

**Fig. 2:**
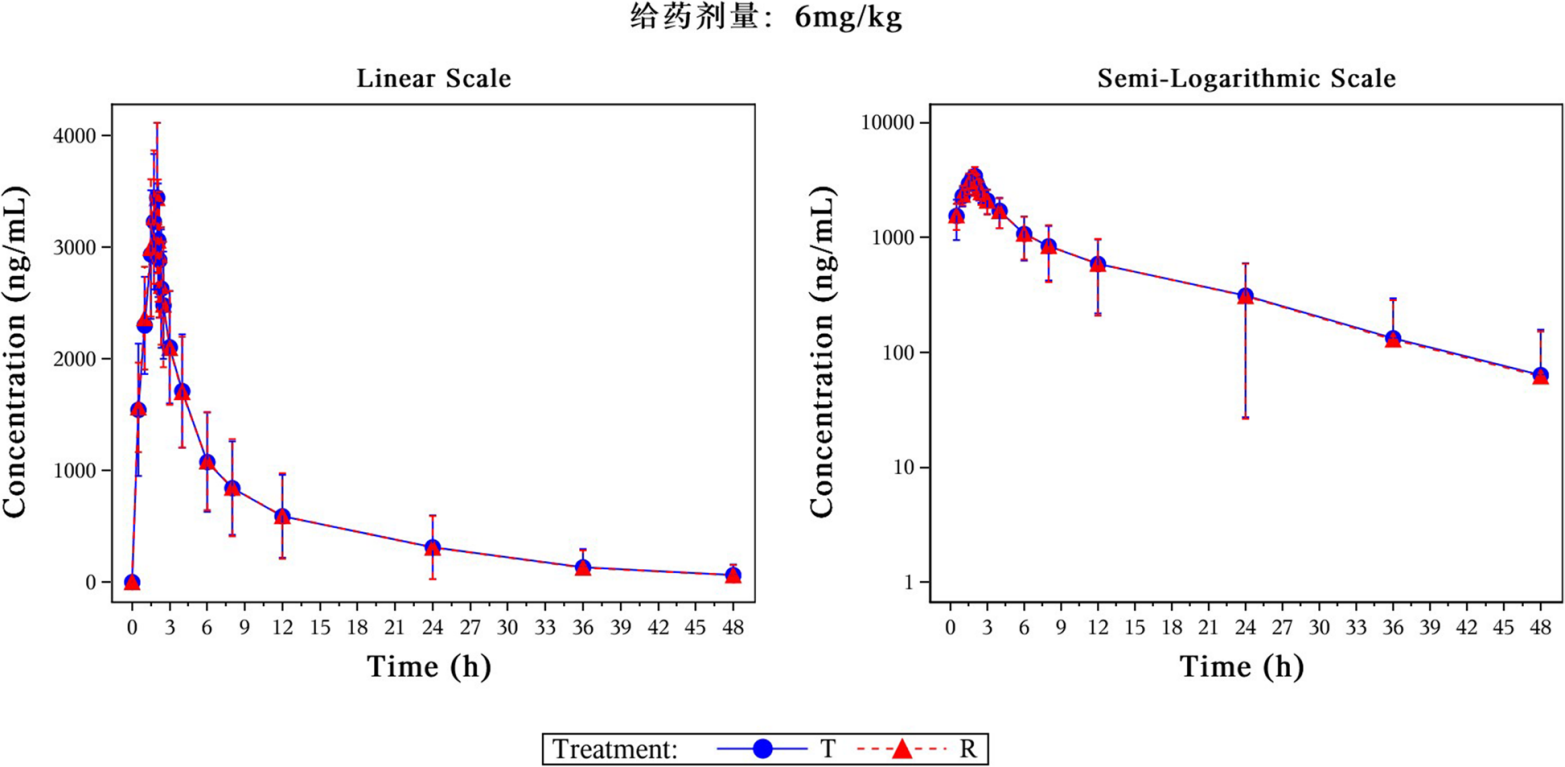
The mean plasma concentration–time curves following a single dose of test formulations or reference formulations in the 6 mg/kg group

Mean pharmacokinetic parameters from the 4 mg/kg group (Table 2) and 6 mg/kg group (Table 3) are summarized. The 90% CIs for the GMRs of C_max_, AUC_0-t_, AUC_0-∞_ and the power were presented in Tables 4 (the 4 mg/kg group) and 5 (the 6 mg/kg group). These ratios were within the predefined equivalence limit of 80 ~ 125%.

**Table 2:** Pharmacokinetic parameters of Voriconazole test and reference formulations in the 4 mg/kg group (*n* = 24)

Parameter	Arithmetic mean ± SD (%CV)^a^	
	T	R
C_max_^b^(μg/mL)	2.552 ± 0.448 (0.018)	2.615 ± 0.464 (0.018)
AUC_0–t_^c^(h*μg/mL)	11.875 ± 7.157 (0.060)	12.500 ± 7.257 (0.058)
AUC_0–∞_^d^(h*μg/mL)	12.835 ± 9.813 (0.076)	13.416 ± 9.485 (0.071)
T_1/2_^e^(h)	7.96 ± 4.89 (61.41)	7.91 ± 4.45 (56.24)
t_max_^f^(h)	1.33 (1.00, 1.50)	1.33 (1.00, 1.45)

^a^All values are represented as arithmetic mean ± standard deviation (CV, %) with [geometric mean] unless otherwise specified. *t_max_ is shown as median (minimum–maximum)

^b^C_max_: maximum plasma concentration

^c^AUC_0-t_: area under the plasma concentration–time curve from 0 to the last measured time point

^d^AUC_0-∞_: area under the plasma concentration–time curve from 0 to infinity

^e^T_1/2_: terminal half-life

^f^T_max_: time to C_max_

**Table 3:** Pharmacokinetic parameters of Voriconazole test and reference formulations in the 6 mg/kg group (*n* = 24)

Parameter	Arithmetic mean ± SD (%CV)	
	T	R
C_max_(μg/mL)	3.538 ± 0.691(0.020)	3.504 ± 0.667(0.019)
AUC_0–t_(h*μg/mL)	24.976 ± 12.364(0.050)	24.990 ± 12.455(0.050)
AUC_0–∞_(h*μg/mL)	26.212 ± 14.057(0.054)	26.160 ± 13.996(0.054)
T_1/2_ (h)	8.65 ± 3.06(35.37)	8.44 ± 2.98(35.35)
t_max_ (h)	2.00(1.75, 2.08)	2.00(1.50, 2.17)

**Table 4:** 90% CIs for the geometric mean ratios of C_max_, AUC_0–t_, and AUC_0–∞_ in the 4 mg/kg group (*n* = 24)

Parameter	T/R (%)	90% CIs	Power (%)
C_max_	97.55	(94.40,100.80)	> 99.9
AUC_0–t_	94.63	(92.41,96.90)	> 99.9
AUC_0–∞_	94.59	(92.20,97.04)	> 99.9

**Table 5:** 90% CIs for the geometric mean ratios of C_max_, AUC_0–t_, and AUC_0–∞_ in the 6 mg/kg group (*n* = 24)

Parameter	T/R (%)	90% CIs	Power (%)
C_max_	100.89	(96.97,104.98)	> 99.9
AUC_0–t_	100.37	(97.70,103.10)	> 99.9
AUC_0–∞_	100.53	(97.77,103.36)	> 99.9

### Safety

During the whole study period, both test preparation and reference preparation showed good safety. The AEs found in physical examination, 12-lead ECG and laboratory examination were listed in Tables 6 (the 4 mg/kg group) and 7 (the 6 mg/kg group). None of them were judged as serious adverse events (SAEs).

**Table 6:** Adverse events in the 4 mg/kg group

Random number	AEs	Treatment	Relationship with the formulations
1003	hypotension	T	Probably related
	hypotension	R	Probably related
	Ventricular premature beat	R	Possibly related
1004	Urinary tract infection	R	Possibly related
1005	Chest pain	R	may be uncorrelated
1007	T wave change	R	Probably related
	T wave change	T	Probably related
1009	ST segment change	T	Possibly related
1020	T wave change	T	Possibly related
1021	Atrial premature beat	T	Possibly related

**Table 7:** Adverse events in the 6 mg/kg group

Random number	AEs	Treatment	Relationship with the formulations
2001	Atrial premature beat	T	Possibly related
2014	Atrial premature beat	T	Possibly related
2020	New onset atrial block	T	Possibly related
2023	hypotension	R	Probably related

## Discussion

In order to preliminarily verify the pharmacokinetic characteristics and safety of the test formulation, two doses of 4 mg/kg and 6 mg/kg were selected in the clinical dose range of the reference formulation for an open, randomized, two cycle and crossover comparative study.

Invasive fungal infection refers to fungal infection that not only invades the skin and subcutaneous tissue, but also involves deep tissues and organs. Common fungi include Candida, Cryptococcus and Aspergillus [5]. In recent years, with the increase of immunosuppressive therapy, chemotherapy and organ transplantation, and the development of interventional therapy such as central venous catheterization and heart prosthetic valve implantation, the number of patients with invasive fungal infection has increased year by year [6–8]. At present, triazole, polyene, fluorocytosine and echinocandin are the main anti-invasive fungal infection drugs used in clinic.Triazole antifungal drugs are widely used in the treatment of invasive fungal infection because of their wide antifungal spectrum, low toxicity and good safety [9].

Triazole drugs mainly inhibits sterol 14α-sterol demethylase activityby binding with fungal cytochrome P-450, resulting in the accumulation of 14α-methylated sterols and interfering with the synthesis of cell membrane lipids, which destroys the structure and function of cell membrane,and finally leads to the death of fungal cells [2]. The first generation of triazole drugs (fluconazole in 1990 and itraconazole in 1992) showed strong activity against candidiasis, but the effect of treating invasive filamentous fungal diseases (such as aspergillosis and mucormycosis) was not as good as amphotericin B [10]. The second generation of triazole antifungal drugs (Voriconazole and Posaconazole) enhanced the activity against filamentous fungal infection [11]. The concentration of Voriconazole in cerebrospinal fluid is about 50% higher than that in serum, so it can be used to treat the infection of central nervous system [12]. Voriconazole is mainly metabolized by CYP2C19 enzyme. In view of gene polymorphism, the serum drug concentration varies greatly among individuals [13]. Voriconazole is mainly used in the first-line treatment of invasive aspergillosis and in the prevention and treatment of invasive fungal infection in patients undergoing hematopoietic stem cell transplantation [14].

Although high individual variability in Voriconazole pharmacokinetics have been observed and the therapeutic range is relatively narrow, the metabolism of Voriconazole has not been fully elucidated yet. In addition, the nonlinear pharmacokinetics caused by self inhibition or induction and the polymorphism of metabolic enzymes impede the safe and effective administration of Voriconazole in clinic [15]. Following oral administration, Voriconazole is rapidly and almost completely absorbed, with C_max_ occurring 1–2 h after dosing. The absolute bioavailability in adults is estimated to be 96%, thus a switch between the intravenous and oral administration is feasible [16, 17]. In the 4 mg/kg group, the %AUC_ex_ (first cycle and second cycle) of subjects with random number 1018 was greater than 20%, and the terminal phase elimination rate λ_z_ cannot be accurately estimated, so the PK parameters of the subject are excluded in this test(λ_z_, AUC_0-∞_, T_1/2_, Cl, V_Z_, %AUC_ex_ and AUC _0-t_).

Triazole drugs are well tolerated. The common adverse reactions are rash, headache, gastrointestinal reaction, QT abnormality, liver injury, and occasionally liver failure. In addition, Voriconazole may also produce some unique adverse reactions, such as mental disorders (hallucinations, delirium), reversible visual changes (hallucinations, diplopia) and osteotoxicity [18]. In addition, triazole drugs are CYP450 enzyme inhibitors, which can reduce the metabolism of combined drugs and increase the risk of toxicity [19]. In this study, no suspicious unexpected serious adverse reactions and serious adverse events occurred, and it is considered that the safety between the test formulation and the reference formulation are comparable. However, due to the limitations of the study design, the current study results cannot be extended to the older (> 45 years old) patient population.

## Conclusions

The trial confirmed that Voriconazole at 4 mg/kg and 6 mg/kg doses were bioequivalent.

## Supplementary Information

**Additional file 1:** **Table 1. **Individual plasmaconcentration-time data of Voriconazole test formulation in the 4 mg/kg group(ng/ml). **Table 2. **Individual plasma concentration-time data of Voriconazole reference formulation in the 4 mg/kg group (ng/ml). **Table 3. **Individual plasma concentration-time data of Voriconazole test formulation in the 6 mg/kg group (ng/ml). **Table 4. **Individual plasma concentration-time data of Voriconazole reference formulation in the 6 mg/kg group (ng/ml). 

**Additional file 2. **CONSORT 2010 checklist of information to include when reporting a randomised trial.

## Data Availability

We have shared the raw data by providing it in a supplementary file.

## Abbreviations

LC–MS/MS: Liquid chromatography-tandem mass spectrometry
CIs: Confidence intervals
GMRs: Ratio of geometric means
SAE: Serious adverse event
GCP: Good clinical practice
NMPA: National Medical Products Administration
HIV: Human immunodeficiency virus
ECG: Electrocardiogram
AEs: Adverse events
PK: Pharmacokinetic
C_max_: Maximum plasma concentration
AUC_0-t_: Area under the plasma concentration–time curve from 0 to the last measured time point
AUC_0-∞_: Area under the plasma concentration–time curve from 0 to infinity
T_max_: Time to C_max_
T_1/2_: Terminal half-life
ANOVA: Analysis of variance
SD: Standard deviation
