# Supplementary material for: Pharmacokinetics and safety of two Voriconazole formulations after intravenous infusion in two doses in healthy Chinese subjects
Source: BMC Pharmacol Toxicol. 2023 Mar 3;24:14. doi: 10.1186/s40360-023-00652-3 (PMC9985189; doi:10.1186/s40360-023-00652-3)
Supplement: Supplementary file 1 — Additional file 1: Table 1. Individual plasmaconcentration-time data of Voriconazole test formulation in the 4 mg/kg group(ng/ml). Table 2. Individual plasma concentration-time data of Voriconazole reference formulation in the 4 mg/kg group (ng/ml). Table 3. Individual plasma concentration-time data of Voriconazole test formulation in the 6 mg/kg group (ng/ml). Table 4. Individual plasma concentration-time data of Voriconazole reference formulation in the 6 mg/kg group (ng/ml). [file 40360_2023_652_MOESM1_ESM.docx]

**The 4 mg/kg Group**

**Table 1.** Individual plasma concentration-time data of Voriconazole test formulation in the 4 mg/kg group (ng/ml). (Supplementary file for Fig. 1)

| SUB | SEQ | PER | GRP | TRT | C1 | C2 | C3 | C4 | C5 | C6 | C7 | C8 | C9 |
| --- | --- | --- | --- | --- | --- | --- | --- | --- | --- | --- | --- | --- | --- |
| 1001 | RT | 2 | B | T | BQL | 1730.132 | 2268.452 | 2360.089 | 2232.174 | 1946.487 | 1568.355 | 1363.959 | 1105.207 |
| 1002 | TR | 1 | A | T | BQL | 1886.564 | 2741.267 | 3159.317 | 2400.252 | 2060.023 | 1534.812 | 1312.168 | 1084.184 |
| 1003 | TR | 1 | A | T | BQL | 1239.692 | 1955.918 | 2345.709 | 2142.560 | 1860.904 | 1479.687 | 1277.500 | 1127.370 |
| 1004 | TR | 1 | A | T | BQL | 1542.377 | 2177.631 | 2334.711 | 1837.875 | 1553.172 | 1282.567 | 1132.337 | 989.114 |
| 1005 | RT | 2 | B | T | BQL | 1655.812 | 1983.626 | 2416.245 | 2007.140 | 1682.557 | 1319.212 | 1235.267 | 811.777 |
| 1006 | RT | 2 | B | T | BQL | 1954.229 | 2202.139 | 2773.829 | 2356.391 | 2038.095 | 1754.702 | 1602.965 | 1193.989 |
| 1007 | RT | 2 | B | T | BQL | 1473.531 | 2804.567 | 2760.128 | 2822.353 | 2601.839 | 2264.519 | 2122.587 | 1563.381 |
| 1008 | TR | 1 | A | T | BQL | 2330.192 | 3017.718 | 3103.519 | 2734.155 | 2286.162 | 1822.054 | 1662.885 | 1569.522 |
| 1009 | TR | 1 | A | T | BQL | 688.579 | 1199.262 | 1590.446 | / | 1593.777 | 1328.964 | 1130.846 | 874.376 |
| 1010 | TR | 1 | A | T | BQL | 1638.847 | 2050.253 | 1994.565 | 1961.354 | 1655.051 | 1475.816 | 1330.241 | 1069.913 |
| 1011 | RT | 2 | B | T | BQL | 1059.706 | 1400.304 | 1737.022 | 1473.975 | 1288.157 | 1076.636 | 922.768 | 766.393 |
| 1012 | RT | 2 | B | T | BQL | 1515.239 | 2134.067 | 2536.099 | 2405.079 | 2196.192 | 1853.398 | 1679.194 | 1435.870 |
| 1013 | RT | 2 | B | T | BQL | 1550.264 | 2374.754 | 2436.614 | 2202.278 | 1980.864 | 1660.656 | 1364.346 | 1102.581 |
| 1014 | TR | 1 | A | T | BQL | 2144.146 | 2421.267 | 2944.951 | 2559.827 | 1990.623 | 1652.355 | 1543.523 | 1237.692 |
| 1015 | RT | 2 | B | T | BQL | 1466.135 | 1889.126 | 2723.274 | 2189.531 | 1976.420 | 1615.991 | 1363.560 | 1186.219 |
| 1016 | TR | 1 | A | T | BQL | 913.244 | 1769.949 | 2026.418 | 2089.708 | 1992.488 | 1787.065 | 1587.983 | 1323.259 |
| 1017 | RT | 2 | B | T | BQL | 2196.859 | 2694.811 | 2434.515 | 2029.461 | 1794.131 | 1556.917 | 1295.494 | 1074.976 |
| 1018 | TR | 1 | A | T | BQL | 1804.764 | 2409.603 | 2666.839 | 2993.542 | 2323.053 | 2196.455 | 2152.651 | 1985.527 |
| 1019 | RT | 2 | B | T | BQL | 1591.164 | 2452.976 | 2471.186 | 1960.433 | 1711.698 | 1529.651 | 1338.953 | 978.372 |
| 1020 | TR | 1 | A | T | BQL | 2195.827 | 2985.623 | 2724.877 | 1870.385 | 1820.875 | 1659.668 | 1500.348 | 1341.374 |
| 1021 | RT | 2 | B | T | BQL | 1734.247 | 2189.019 | 2342.778 | 2352.762 | 2191.805 | 1968.958 | 1736.627 | 1451.134 |
| 1022 | TR | 1 | A | T | BQL | 1312.405 | 1666.828 | 2042.242 | 2063.025 | 1919.782 | 1605.132 | 1514.594 | 1199.281 |
| 1023 | TR | 1 | A | T | BQL | 2680.173 | 3148.382 | 3295.864 | 2505.716 | 2311.984 | 1823.757 | 1578.993 | 1354.550 |
| 1024 | RT | 2 | B | T | BQL | 2145.061 | 2776.398 | 2975.375 | 2445.891 | 2258.897 | 1822.065 | 1586.829 | 1286.764 |
| n | | | | | 24 | 24 | 24 | 24 | 23 | 24 | 24 | 24 | 24 |
| Mean | | | | | 0.0000 | 1685.3829 | 2279.7475 | 2508.1922 | 2245.0377 | 1959.7932 | 1651.6413 | 1472.3591 | 1213.0344 |
| SD | | | | | 0.0000 | 464.9579 | 502.2108 | 434.2498 | 347.1930 | 297.2930 | 273.5027 | 284.0480 | 269.9907 |
| %CV | | | | | NA | 27.59 | 22.03 | 17.31 | 15.46 | 15.17 | 16.56 | 19.29 | 22.26 |
| Min | | | | | 0.000 | 688.579 | 1199.262 | 1590.446 | 1473.975 | 1288.157 | 1076.636 | 922.768 | 766.393 |
| Max | | | | | 0.000 | 2680.173 | 3148.382 | 3295.864 | 2993.542 | 2601.839 | 2264.519 | 2152.651 | 1985.527 |

| SUB | SEQ | PER | GRP | TRT | C10 | C11 | C12 | C13 | C14 | C15 | C16 | C17 |
| --- | --- | --- | --- | --- | --- | --- | --- | --- | --- | --- | --- | --- |
| 1001 | RT | 2 | B | T | 926.302 | 718.309 | 352.737 | 187.977 | 129.428 | 37.707 | BQL | BQL |
| 1002 | TR | 1 | A | T | 768.316 | 635.692 | 272.394 | 182.672 | 96.650 | 24.766 | BQL | BQL |
| 1003 | TR | 1 | A | T | 833.139 | 647.315 | 341.339 | 254.072 | 138.149 | 33.581 | BQL | BQL |
| 1004 | TR | 1 | A | T | 823.601 | 614.622 | 333.463 | 269.104 | 159.908 | 48.028 | 11.469 | BQL |
| 1005 | RT | 2 | B | T | 780.555 | 464.567 | 225.038 | 114.700 | 55.347 | 12.183 | BQL | BQL |
| 1006 | RT | 2 | B | T | 923.938 | 737.287 | 382.199 | 269.807 | 130.279 | 38.486 | 11.702 | BQL |
| 1007 | RT | 2 | B | T | 1314.032 | 1097.227 | 770.571 | 624.950 | 488.805 | 283.896 | 116.115 | 55.643 |
| 1008 | TR | 1 | A | T | 1345.665 | 1241.184 | 769.597 | 651.178 | 526.608 | 327.687 | 158.294 | 80.943 |
| 1009 | TR | 1 | A | T | 755.716 | 543.639 | 215.889 | 136.977 | 63.885 | 18.441 | BQL | BQL |
| 1010 | TR | 1 | A | T | 934.424 | 659.982 | 390.087 | 218.249 | 96.409 | 22.316 | BQL | BQL |
| 1011 | RT | 2 | B | T | 453.023 | 468.336 | 261.518 | 171.337 | 71.761 | 14.725 | BQL | BQL |
| 1012 | RT | 2 | B | T | 1275.376 | 1103.181 | 814.741 | 629.874 | 462.371 | 238.979 | 103.099 | 45.723 |
| 1013 | RT | 2 | B | T | 941.361 | 572.009 | 247.358 | 156.533 | 92.131 | 38.173 | BQL | BQL |
| 1014 | TR | 1 | A | T | 1137.498 | 937.373 | 494.833 | 346.631 | 232.372 | 127.579 | 27.683 | 13.986 |
| 1015 | RT | 2 | B | T | 1024.057 | 825.017 | 466.878 | 350.155 | 215.359 | 78.804 | 22.268 | BQL |
| 1016 | TR | 1 | A | T | 1163.455 | 986.149 | 582.202 | 305.533 | 189.267 | 89.259 | 17.850 | BQL |
| 1017 | RT | 2 | B | T | 972.999 | 718.235 | 411.123 | 225.286 | 109.393 | 26.061 | BQL | BQL |
| 1018 | TR | 1 | A | T | 1673.800 | 1504.487 | 1070.702 | 996.778 | 861.674 | 738.608 | 475.012 | 375.103 |
| 1019 | RT | 2 | B | T | 859.188 | 588.789 | 262.376 | 188.139 | 92.089 | 31.703 | BQL | BQL |
| 1020 | TR | 1 | A | T | 1169.566 | 1069.003 | 724.567 | 536.124 | 420.873 | 253.759 | 117.779 | 65.208 |
| 1021 | RT | 2 | B | T | 1341.283 | 1085.329 | 658.548 | 480.611 | 253.472 | 65.922 | 10.602 | BQL |
| 1022 | TR | 1 | A | T | 1014.540 | 786.833 | 374.794 | 229.607 | 86.990 | 35.038 | BQL | BQL |
| 1023 | TR | 1 | A | T | 988.742 | 782.529 | 340.544 | 283.211 | 157.111 | 76.945 | 19.124 | BQL |
| 1024 | RT | 2 | B | T | 1166.286 | 836.964 | 481.440 | 341.446 | 208.874 | 74.577 | 18.067 | BQL |
| n | | | | | 24 | 24 | 24 | 24 | 24 | 24 | 24 | 24 |
| Mean | | | | | 1024.4526 | 817.6691 | 468.5391 | 339.6230 | 222.4669 | 114.0510 | 46.2110 | 26.5253 |
| SD | | | | | 256.4109 | 262.0888 | 225.1126 | 212.5133 | 195.2061 | 161.7357 | 102.1679 | 78.0196 |
| %CV | | | | | 25.03 | 32.05 | 48.05 | 62.57 | 87.75 | 141.81 | 221.09 | 294.13 |
| Min | | | | | 453.023 | 464.567 | 215.889 | 114.700 | 55.347 | 12.183 | 0.000 | 0.000 |
| Max | | | | | 1673.800 | 1504.487 | 1070.702 | 996.778 | 861.674 | 738.608 | 475.012 | 375.103 |

**Table 2.** Individual plasma concentration-time data of Voriconazole reference formulation in the 4 mg/kg group (ng/ml). (Supplementary file for Fig. 1)

| SUB | SEQ | PER | GRP | TRT | C1 | C2 | C3 | C4 | C5 | C6 | C7 | C8 | C9 |
| --- | --- | --- | --- | --- | --- | --- | --- | --- | --- | --- | --- | --- | --- |
| 1001 | RT | 1 | B | R | BQL | 1918.223 | 2574.696 | 2416.837 | 2307.755 | 1822.289 | 1388.263 | 1304.809 | 1161.689 |
| 1002 | TR | 2 | A | R | BQL | 2254.041 | 2807.728 | 3000.856 | 2413.022 | 2088.763 | 1711.596 | 1557.952 | 1179.332 |
| 1003 | TR | 2 | A | R | BQL | 1332.931 | 2103.800 | 2491.388 | 2294.913 | 2065.226 | 1515.549 | 1511.380 | 1156.460 |
| 1004 | TR | 2 | A | R | BQL | 1947.538 | 2569.486 | 2413.626 | 2057.693 | 1853.492 | 1560.869 | 1406.655 | 1077.867 |
| 1005 | RT | 1 | B | R | BQL | 1448.859 | 2176.706 | 2148.199 | 1791.027 | 1506.748 | 1183.125 | 932.977 | 920.921 |
| 1006 | RT | 1 | B | R | BQL | 1706.663 | 2252.998 | 2455.295 | 2108.084 | 1916.851 | 1654.863 | 1283.446 | 1161.571 |
| 1007 | RT | 1 | B | R | BQL | 1902.804 | 3260.765 | 2647.755 | 2839.380 | 2522.244 | 2173.108 | 1951.647 | 1600.505 |
| 1008 | TR | 2 | A | R | BQL | 2497.292 | 3311.806 | 3286.335 | 2837.831 | 2434.909 | 2007.351 | 1851.986 | 1512.290 |
| 1009 | TR | 2 | A | R | BQL | 809.403 | 1415.047 | 1748.168 | / | 1689.981 | 1363.013 | 1156.956 | 764.472 |
| 1010 | TR | 2 | A | R | BQL | 1663.986 | 2110.029 | 2153.563 | 1929.284 | 1763.258 | 1576.325 | 1407.011 | 1143.671 |
| 1011 | RT | 1 | B | R | BQL | 1345.664 | 1367.020 | 1772.953 | 1468.179 | 1183.922 | 978.690 | 816.755 | 726.892 |
| 1012 | RT | 1 | B | R | BQL | 1286.390 | 2322.439 | 2476.408 | 2057.128 | 2070.767 | 1784.951 | 1621.861 | 1363.908 |
| 1013 | RT | 1 | B | R | BQL | 1853.016 | 2721.885 | 2272.786 | 2151.161 | 1833.036 | 1447.464 | 1316.677 | 965.395 |
| 1014 | TR | 2 | A | R | BQL | 1305.489 | 1589.642 | 2658.000 | 2637.637 | 2107.150 | 1853.451 | 1582.030 | 1276.169 |
| 1015 | RT | 1 | B | R | BQL | 1752.286 | 2403.296 | 2140.158 | 2293.974 | 1960.380 | 1521.795 | 1451.916 | 1095.142 |
| 1016 | TR | 2 | A | R | BQL | 761.392 | 1661.110 | 2290.718 | 1945.470 | 2099.381 | 1736.907 | 1481.942 | 1272.607 |
| 1017 | RT | 1 | B | R | BQL | 1885.752 | 2592.999 | 2255.125 | 1933.936 | 1831.621 | 1475.873 | 1328.604 | 1084.671 |
| 1018 | TR | 2 | A | R | BQL | 2074.246 | 2976.669 | 3319.879 | 2578.755 | 2527.285 | 2153.259 | 2097.604 | 1702.980 |
| 1019 | RT | 1 | B | R | BQL | 1514.001 | 1943.535 | 2447.104 | 2040.403 | 1787.471 | 1511.314 | 1429.311 | 1046.363 |
| 1020 | TR | 2 | A | R | BQL | 2433.188 | 3525.321 | 3383.738 | 2660.857 | 2552.302 | 2161.190 | 1830.114 | 1576.921 |
| 1021 | RT | 1 | B | R | BQL | 1648.940 | 2348.500 | 2415.199 | 2558.444 | 2391.334 | 2162.384 | 1967.822 | 1646.898 |
| 1022 | TR | 2 | A | R | BQL | 1958.656 | 2060.052 | 2271.304 | 2102.878 | 1949.923 | 1609.565 | 1472.645 | 1101.792 |
| 1023 | TR | 2 | A | R | BQL | 2518.127 | 2831.262 | 2733.655 | 2442.938 | 2184.797 | 1933.730 | 1675.126 | 1242.899 |
| 1024 | RT | 1 | B | R | BQL | 2377.561 | 3119.234 | 3156.411 | 2592.705 | 2238.737 | 1794.983 | 1580.799 | 1310.496 |
| n | | | | | 24 | 24 | 24 | 24 | 23 | 24 | 24 | 24 | 24 |
| Mean | | | | | 0.0000 | 1758.1853 | 2418.5844 | 2514.8108 | 2262.7589 | 2015.9111 | 1677.4841 | 1500.7510 | 1212.1630 |
| SD | | | | | 0.0000 | 480.3884 | 591.5437 | 444.8340 | 349.4833 | 332.8752 | 315.7066 | 306.0857 | 257.7905 |
| %CV | | | | | NA | 27.32 | 24.46 | 17.69 | 15.45 | 16.51 | 18.82 | 20.40 | 21.27 |
| Min | | | | | 0.000 | 761.392 | 1367.020 | 1748.168 | 1468.179 | 1183.922 | 978.690 | 816.755 | 726.892 |
| Max | | | | | 0.000 | 2518.127 | 3525.321 | 3383.738 | 2839.380 | 2552.302 | 2173.108 | 2097.604 | 1702.980 |

| SUB | SEQ | PER | GRP | TRT | C10 | C11 | C12 | C13 | C14 | C15 | C16 | C17 |
| --- | --- | --- | --- | --- | --- | --- | --- | --- | --- | --- | --- | --- |
| 1001 | RT | 1 | B | R | 908.166 | 594.031 | 323.585 | 226.086 | 120.510 | 46.339 | 10.132 | BQL |
| 1002 | TR | 2 | A | R | 931.901 | 719.828 | 330.058 | 211.283 | 135.984 | 42.304 | BQL | BQL |
| 1003 | TR | 2 | A | R | 913.529 | 674.019 | 371.610 | 290.877 | 157.437 | 51.199 | BQL | BQL |
| 1004 | TR | 2 | A | R | 957.101 | 807.066 | 476.860 | 372.066 | 207.990 | 73.290 | 21.734 | BQL |
| 1005 | RT | 1 | B | R | 719.492 | 508.453 | 202.676 | 143.083 | 59.778 | 15.662 | BQL | BQL |
| 1006 | RT | 1 | B | R | 1046.601 | 713.414 | 388.536 | 336.742 | 122.010 | 38.490 | BQL | BQL |
| 1007 | RT | 1 | B | R | 1421.319 | 1124.924 | 788.827 | 654.002 | 498.534 | 271.496 | 110.878 | 50.868 |
| 1008 | TR | 2 | A | R | 1323.267 | 1118.650 | 822.709 | 696.775 | 541.983 | 376.649 | 182.454 | 106.976 |
| 1009 | TR | 2 | A | R | 743.903 | 516.493 | 257.040 | 158.559 | 70.764 | 18.987 | BQL | BQL |
| 1010 | TR | 2 | A | R | 1046.530 | 788.438 | 505.719 | 348.419 | 147.977 | 39.693 | BQL | BQL |
| 1011 | RT | 1 | B | R | 538.000 | 502.157 | 266.195 | 147.513 | 79.467 | 16.981 | BQL | BQL |
| 1012 | RT | 1 | B | R | 1207.149 | 1085.115 | 709.384 | 565.907 | 421.669 | 222.174 | 81.807 | 31.867 |
| 1013 | RT | 1 | B | R | 730.660 | 695.250 | 279.921 | 189.044 | 110.413 | 41.807 | BQL | BQL |
| 1014 | TR | 2 | A | R | 1181.094 | 924.630 | 660.223 | 481.816 | 290.517 | 122.868 | 41.401 | 20.109 |
| 1015 | RT | 1 | B | R | 961.739 | 785.837 | 454.628 | 333.022 | 193.533 | 90.997 | 17.759 | BQL |
| 1016 | TR | 2 | A | R | 1176.970 | 1062.560 | 527.767 | 366.512 | 235.526 | 91.663 | 24.781 | BQL |
| 1017 | RT | 1 | B | R | 935.676 | 687.442 | 368.269 | 219.417 | 105.018 | 26.835 | BQL | BQL |
| 1018 | TR | 2 | A | R | 1607.857 | 1447.694 | 1130.817 | 1011.999 | 864.925 | 729.486 | 463.747 | 348.018 |
| 1019 | RT | 1 | B | R | 931.841 | 648.858 | 281.048 | 191.862 | 113.759 | 29.841 | BQL | BQL |
| 1020 | TR | 2 | A | R | 1420.728 | 1143.597 | 809.677 | 714.747 | 516.807 | 288.847 | 141.733 | 72.843 |
| 1021 | RT | 1 | B | R | 1457.763 | 1262.764 | 770.760 | 594.060 | 326.175 | 99.391 | 22.971 | BQL |
| 1022 | TR | 2 | A | R | 1080.867 | 883.270 | 371.306 | 258.244 | 99.691 | 23.832 | BQL | BQL |
| 1023 | TR | 2 | A | R | 1079.636 | 796.066 | 429.182 | 329.757 | 212.234 | 90.871 | 19.630 | BQL |
| 1024 | RT | 1 | B | R | 1084.110 | 873.374 | 465.703 | 314.286 | 172.834 | 81.094 | 19.784 | BQL |
| n | | | | | 24 | 24 | 24 | 24 | 24 | 24 | 24 | 24 |
| Mean | | | | | 1058.5791 | 848.4971 | 499.6875 | 381.5033 | 241.8973 | 122.1165 | 48.2838 | 26.2784 |
| SD | | | | | 259.5912 | 251.4007 | 233.6013 | 220.8231 | 196.5352 | 161.3689 | 101.2952 | 73.8096 |
| %CV | | | | | 24.52 | 29.63 | 46.75 | 57.88 | 81.25 | 132.14 | 209.79 | 280.88 |
| Min | | | | | 538.000 | 502.157 | 202.676 | 143.083 | 59.778 | 15.662 | 0.000 | 0.000 |
| Max | | | | | 1607.857 | 1447.694 | 1130.817 | 1011.999 | 864.925 | 729.486 | 463.747 | 348.018 |

The concentration lower than the lower limit of quantification before T_max_ was expressed as 0. The concentration lower than the lower limit of quantification after T_max_ was expressed as deletion (ND) in PK analysis and 0 in descriptive statistics.

**The 6 mg/kg Group**

**Table 3.** Individual plasma concentration-time data of Voriconazole test formulation in the 6 mg/kg group (ng/ml). (Supplementary file for Fig. 2)

| SUB | SEQ | PER | GRP | TRT | C1 | C2 | C3 | C4 | C5 | C6 | C7 | C8 | C9 |
| --- | --- | --- | --- | --- | --- | --- | --- | --- | --- | --- | --- | --- | --- |
| 2001 | TR | 1 | A | T | BQL | 1729.760 | 2696.489 | 3207.809 | 3543.988 | 3501.201 | 3282.268 | 2977.044 | 2751.522 |
| 2002 | TR | 1 | A | T | BQL | 1410.075 | 2053.950 | 3016.871 | 3423.470 | 4144.189 | 4017.833 | 3570.189 | 3352.761 |
| 2003 | RT | 2 | B | T | BQL | 935.976 | 2116.825 | 2679.653 | 2831.708 | 3130.844 | 2588.675 | 2225.099 | 2004.539 |
| 2004 | RT | 2 | B | T | BQL | 1442.549 | 1726.166 | 2180.657 | 2541.663 | 2760.833 | 2376.466 | 2136.965 | 1817.940 |
| 2005 | RT | 2 | B | T | BQL | 1590.005 | 1824.947 | 3205.286 | 3789.205 | 3189.935 | 2979.185 | 2781.196 | 2639.221 |
| 2006 | TR | 1 | A | T | BQL | 1059.607 | 2307.762 | 2840.910 | 4036.093 | 4600.141 | 4174.313 | 3788.416 | 3431.542 |
| 2007 | RT | 2 | B | T | BQL | 867.460 | 1557.052 | 2162.804 | 2383.687 | 2961.548 | 2918.773 | 2790.436 | 2569.549 |
| 2008 | RT | 2 | B | T | BQL | 1925.804 | 2677.702 | 3213.792 | 3534.716 | 3181.963 | 3339.853 | 3102.140 | 2789.361 |
| 2009 | TR | 1 | A | T | BQL | 1950.907 | 2599.194 | 3043.540 | 3209.994 | 2853.405 | 3009.584 | 2651.015 | 2274.579 |
| 2010 | RT | 2 | B | T | BQL | 1952.393 | 2529.575 | 3211.709 | 3184.433 | 3741.838 | 2846.054 | 2650.540 | 2242.249 |
| 2011 | TR | 1 | A | T | BQL | 1126.374 | 2186.997 | 2284.592 | 2360.319 | 2531.251 | 2432.274 | 2226.877 | 1932.424 |
| 2012 | TR | 1 | A | T | BQL | 1066.166 | 1811.918 | 2166.965 | 2396.587 | 2443.598 | 2378.780 | 2292.024 | 1926.979 |
| 2013 | TR | 1 | A | T | BQL | 1260.143 | 1852.065 | 2187.511 | 2459.665 | 2763.686 | 2250.967 | 2172.192 | 1891.113 |
| 2014 | TR | 1 | A | T | BQL | 1009.559 | 2688.525 | 2946.376 | 2845.318 | 3505.034 | 3494.956 | 3140.206 | 2951.294 |
| 2015 | RT | 2 | B | T | BQL | 1657.000 | 2513.476 | 4180.167 | 3784.571 | 4893.071 | 3314.152 | 3719.388 | 3310.298 |
| 2016 | TR | 1 | A | T | BQL | 1068.355 | 2988.484 | 3641.164 | 3990.058 | 4703.551 | 3660.603 | 3499.298 | 3135.810 |
| 2017 | RT | 2 | B | T | BQL | 1751.251 | 2375.499 | 2982.631 | 3514.305 | 3862.532 | 3356.737 | 3416.652 | 3097.147 |
| 2018 | RT | 2 | B | T | BQL | 1689.874 | 2245.577 | 2856.717 | 3512.704 | 3763.419 | 3261.456 | 3145.146 | 2854.900 |
| 2019 | RT | 2 | B | T | BQL | 1613.223 | 1909.809 | 3016.923 | 3423.951 | 3432.891 | 3211.353 | 3049.975 | 3193.629 |
| 2020 | TR | 1 | A | T | BQL | 1862.979 | 2770.613 | 3136.477 | 3331.554 | 3525.164 | 2641.422 | 2460.675 | 2206.611 |
| 2021 | TR | 1 | A | T | BQL | 3744.125 | 3069.324 | 4141.470 | 4636.316 | 3883.061 | 3306.999 | 3284.892 | 3052.251 |
| 2022 | RT | 2 | B | T | BQL | 1327.384 | 2443.576 | 2960.635 | 2996.726 | 3409.371 | 3344.163 | 3256.846 | 3224.064 |
| 2023 | TR | 1 | A | T | BQL | 1961.272 | 2663.330 | 3200.508 | 3344.988 | 3282.886 | 2672.889 | 2444.042 | 2260.052 |
| 2024 | RT | 2 | B | T | BQL | 1057.450 | 1613.682 | 1998.420 | 2447.869 | 2654.342 | 2672.410 | 2472.319 | 2234.922 |
| n | | | | | 24 | 24 | 24 | 24 | 24 | 24 | 24 | 24 | 24 |
| Mean | | | | | 0.0000 | 1544.1538 | 2300.9390 | 2935.9828 | 3230.1620 | 3446.6564 | 3063.8402 | 2885.5655 | 2631.0315 |
| SD | | | | | 0.0000 | 592.7847 | 435.5519 | 576.3490 | 607.4130 | 669.6020 | 508.4670 | 515.7430 | 530.5711 |
| %CV | | | | | NA | 38.39 | 18.93 | 19.63 | 18.80 | 19.43 | 16.60 | 17.87 | 20.17 |
| Min | | | | | 0.000 | 867.460 | 1557.052 | 1998.420 | 2360.319 | 2443.598 | 2250.967 | 2136.965 | 1817.940 |
| Max | | | | | 0.000 | 3744.125 | 3069.324 | 4180.167 | 4636.316 | 4893.071 | 4174.313 | 3788.416 | 3431.542 |

| SUB | SEQ | PER | GRP | TRT | C10 | C11 | C12 | C13 | C14 | C15 | C16 | C17 | C18 |
| --- | --- | --- | --- | --- | --- | --- | --- | --- | --- | --- | --- | --- | --- |
| 2001 | TR | 1 | A | T | 2644.590 | 2167.686 | 1722.638 | 1023.446 | 764.533 | 475.368 | 170.323 | 52.253 | 18.901 |
| 2002 | TR | 1 | A | T | 3141.833 | 2778.859 | 2292.480 | 1650.038 | 1381.057 | 1002.723 | 613.758 | 234.711 | 113.803 |
| 2003 | RT | 2 | B | T | 1873.626 | 1445.455 | 1057.929 | 539.082 | 340.737 | 274.058 | 84.231 | 19.410 | BQL |
| 2004 | RT | 2 | B | T | 1840.422 | 1465.043 | 1106.435 | 587.364 | 474.502 | 312.910 | 105.398 | 31.267 | BQL |
| 2005 | RT | 2 | B | T | 2451.321 | 2192.812 | 1748.284 | 1034.187 | 744.426 | 420.106 | 166.030 | 55.233 | 17.574 |
| 2006 | TR | 1 | A | T | 3269.900 | 2734.385 | 2473.233 | 1657.387 | 1482.789 | 1039.779 | 683.966 | 244.523 | 95.214 |
| 2007 | RT | 2 | B | T | 2438.044 | 1972.184 | 1616.657 | 725.404 | 505.771 | 258.090 | 83.372 | 16.655 | BQL |
| 2008 | RT | 2 | B | T | 2524.728 | 1991.503 | 1689.328 | 1147.195 | 736.503 | 436.482 | 128.371 | 29.803 | BQL |
| 2009 | TR | 1 | A | T | 2200.682 | 1594.214 | 1249.532 | 733.178 | 647.136 | 401.844 | 210.913 | 74.895 | 33.317 |
| 2010 | RT | 2 | B | T | 2241.945 | 1796.247 | 1150.494 | 606.573 | 419.358 | 194.565 | 39.226 | BQL | BQL |
| 2011 | TR | 1 | A | T | 1867.730 | 1519.065 | 1184.814 | 665.945 | 324.403 | 140.013 | 41.700 | BQL | BQL |
| 2012 | TR | 1 | A | T | 1922.033 | 1599.713 | 1233.659 | 572.731 | 391.960 | 276.839 | 118.407 | 27.687 | 12.609 |
| 2013 | TR | 1 | A | T | 1655.845 | 1359.261 | 1045.542 | 582.243 | 412.699 | 265.846 | 83.189 | 19.570 | BQL |
| 2014 | TR | 1 | A | T | 2488.190 | 2181.400 | 1694.487 | 945.919 | 697.470 | 409.509 | 168.086 | 40.072 | 18.651 |
| 2015 | RT | 2 | B | T | 3063.041 | 2581.528 | 2269.366 | 1427.414 | 1135.905 | 817.747 | 415.245 | 178.885 | 63.569 |
| 2016 | TR | 1 | A | T | 3062.359 | 2710.249 | 2280.627 | 1862.583 | 1565.624 | 1291.693 | 966.571 | 532.145 | 305.820 |
| 2017 | RT | 2 | B | T | 2872.832 | 2792.029 | 2418.508 | 1777.637 | 1489.843 | 1312.082 | 929.471 | 562.016 | 316.760 |
| 2018 | RT | 2 | B | T | 2726.384 | 2254.011 | 1806.518 | 1111.088 | 927.181 | 558.850 | 252.140 | 100.712 | 41.937 |
| 2019 | RT | 2 | B | T | 2907.368 | 2570.045 | 1982.998 | 1233.625 | 896.052 | 564.698 | 175.394 | 42.228 | 13.328 |
| 2020 | TR | 1 | A | T | 1978.931 | 1500.728 | 1024.562 | 494.108 | 327.432 | 197.674 | 88.639 | 18.681 | 10.766 |
| 2021 | TR | 1 | A | T | 2948.199 | 2446.747 | 1974.927 | 1264.002 | 1063.905 | 840.899 | 497.703 | 237.106 | 117.812 |
| 2022 | RT | 2 | B | T | 2999.240 | 2959.886 | 2702.465 | 1842.612 | 1563.591 | 1234.419 | 611.579 | 248.433 | 90.570 |
| 2023 | TR | 1 | A | T | 2219.350 | 1881.627 | 1616.872 | 1187.494 | 1062.881 | 926.848 | 664.076 | 377.719 | 233.581 |
| 2024 | RT | 2 | B | T | 2223.718 | 2033.578 | 1755.815 | 1161.362 | 871.431 | 514.018 | 204.409 | 47.310 | 20.870 |
| n | | | | | 24 | 24 | 24 | 24 | 24 | 24 | 24 | 24 | 24 |
| Mean | | | | | 2481.7630 | 2105.3440 | 1712.4238 | 1076.3590 | 842.7995 | 590.2942 | 312.5915 | 132.9714 | 63.5451 |
| SD | | | | | 480.9753 | 503.2672 | 508.6883 | 446.6801 | 419.3000 | 371.4599 | 285.2814 | 163.3024 | 94.2759 |
| %CV | | | | | 19.38 | 23.90 | 29.71 | 41.50 | 49.75 | 62.93 | 91.26 | 122.81 | 148.36 |
| Min | | | | | 1655.845 | 1359.261 | 1024.562 | 494.108 | 324.403 | 140.013 | 39.226 | 0.000 | 0.000 |
| Max | | | | | 3269.900 | 2959.886 | 2702.465 | 1862.583 | 1565.624 | 1312.082 | 966.571 | 562.016 | 316.760 |

**Table 4.** Individual plasma concentration-time data of Voriconazole reference formulation in the 6 mg/kg group (ng/ml). (Supplementary file for Fig. 2)

| SUB | SEQ | PER | GRP | TRT | C1 | C2 | C3 | C4 | C5 | C6 | C7 | C8 | C9 |
| --- | --- | --- | --- | --- | --- | --- | --- | --- | --- | --- | --- | --- | --- |
| 2001 | TR | 2 | A | R | BQL | 1720.242 | 2504.213 | 3506.685 | 3706.161 | 3961.492 | 3399.726 | 3103.798 | 3008.016 |
| 2002 | TR | 2 | A | R | BQL | 1801.078 | 2827.372 | 3499.928 | 3831.671 | 4056.021 | 3740.736 | 3411.189 | 3142.539 |
| 2003 | RT | 1 | B | R | BQL | 1238.973 | 1602.939 | 2235.928 | 2653.374 | 2841.511 | 2314.710 | 2012.467 | 1792.623 |
| 2004 | RT | 1 | B | R | BQL | 1340.576 | 1936.962 | 2200.695 | 2263.487 | 2459.963 | 2186.552 | 1931.072 | 1633.791 |
| 2005 | RT | 1 | B | R | BQL | 2078.528 | 2114.882 | 2464.517 | 2909.345 | 2938.895 | 3147.737 | 2991.657 | 2686.336 |
| 2006 | TR | 2 | A | R | BQL | 1819.623 | 2259.540 | 3340.676 | 3871.227 | 3893.973 | 3848.778 | 3972.389 | 3430.945 |
| 2007 | RT | 1 | B | R | BQL | 1225.143 | 2175.567 | 2882.672 | 3258.977 | 3059.085 | 2994.781 | 2655.934 | 2493.256 |
| 2008 | RT | 1 | B | R | BQL | 1338.580 | 2369.013 | 3067.406 | 3486.712 | 3556.811 | 3498.988 | 3075.780 | 2765.211 |
| 2009 | TR | 2 | A | R | BQL | 1900.782 | 3183.968 | 3872.679 | 3822.290 | 3698.698 | 3436.699 | 3251.030 | 2844.089 |
| 2010 | RT | 1 | B | R | BQL | 1731.177 | 2595.822 | 2766.876 | 2674.890 | 2593.655 | 2314.649 | 2228.564 | 1949.422 |
| 2011 | TR | 2 | A | R | BQL | 958.800 | 1621.691 | 2319.429 | 2484.021 | 2361.456 | 2327.250 | 2281.190 | 2016.135 |
| 2012 | TR | 2 | A | R | BQL | 1322.344 | 2017.116 | 2098.687 | 2370.229 | 2563.458 | 2603.277 | 2504.419 | 2330.870 |
| 2013 | TR | 2 | A | R | BQL | 1424.524 | 2230.805 | 2698.713 | 2756.529 | 3054.058 | 2257.966 | 2217.070 | 1995.015 |
| 2014 | TR | 2 | A | R | BQL | 1479.341 | 3158.923 | 3633.052 | 4343.280 | 3949.136 | 3344.667 | 3208.745 | 3063.829 |
| 2015 | RT | 1 | B | R | BQL | 1389.250 | 1933.732 | 3188.425 | 3794.674 | 4444.106 | 3752.604 | 3667.319 | 3051.976 |
| 2016 | TR | 2 | A | R | BQL | 2273.989 | 3227.395 | 3661.830 | 4124.625 | 4599.036 | 3494.647 | 3499.349 | 3196.475 |
| 2017 | RT | 1 | B | R | BQL | 1215.693 | 2109.988 | 2679.651 | 3195.262 | 3378.556 | 3174.561 | 3130.853 | 3089.911 |
| 2018 | RT | 1 | B | R | BQL | 1012.161 | 2436.974 | 2682.060 | 3178.072 | 3479.850 | 3191.454 | 3100.184 | 2837.154 |
| 2019 | RT | 1 | B | R | BQL | 1406.965 | 2026.690 | 2631.332 | 2998.905 | 3327.283 | 2949.588 | 3059.693 | 2656.408 |
| 2020 | TR | 2 | A | R | BQL | 1629.277 | 2618.119 | 2634.945 | 3419.364 | 3537.072 | 2643.109 | 2561.382 | 2319.602 |
| 2021 | TR | 2 | A | R | BQL | 2621.383 | 2784.396 | 4633.646 | 4020.318 | 4810.995 | 3872.224 | 3653.170 | 3413.676 |
| 2022 | RT | 1 | B | R | BQL | 1724.852 | 2574.911 | 3261.347 | 3709.709 | 3879.247 | 3534.983 | 3614.009 | 3274.587 |
| 2023 | TR | 2 | A | R | BQL | 1800.766 | 2618.692 | 3352.274 | 2901.684 | 3253.407 | 2769.927 | 2646.526 | 2255.111 |
| 2024 | RT | 1 | B | R | BQL | 1132.540 | 1855.799 | 2565.647 | 2749.062 | 2909.754 | 2666.393 | 2674.090 | 2464.580 |
| n | | | 24 | | | 24 | 24 | 24 | 24 | 24 | 24 | 24 | 24 |
| Mean | | | 0.0000 | | | 1566.1078 | 2366.0629 | 2994.9625 | 3271.8278 | 3441.9799 | 3061.0836 | 2935.4950 | 2654.6482 |
| SD | | | 0.0000 | | | 401.0846 | 460.3670 | 616.0574 | 597.7687 | 674.9089 | 547.7483 | 563.2628 | 526.7222 |
| %CV | | | NA | | | 25.61 | 19.46 | 20.57 | 18.27 | 19.61 | 17.89 | 19.19 | 19.84 |
| Min | | | 0.000 | | | 958.800 | 1602.939 | 2098.687 | 2263.487 | 2361.456 | 2186.552 | 1931.072 | 1633.791 |
| Max | | | 0.000 | | | 2621.383 | 3227.395 | 4633.646 | 4343.280 | 4810.995 | 3872.224 | 3972.389 | 3430.945 |

| SUB | SEQ | PER | GRP | TRT | C10 | C11 | C12 | C13 | C14 | C15 | C16 | C17 | C18 |
| --- | --- | --- | --- | --- | --- | --- | --- | --- | --- | --- | --- | --- | --- |
| 2001 | TR | 2 | A | R | 2621.438 | 2326.742 | 1737.610 | 1076.352 | 788.388 | 481.158 | 217.875 | 60.560 | 24.657 |
| 2002 | TR | 2 | A | R | 2898.668 | 2565.751 | 2131.473 | 1436.786 | 1301.744 | 976.664 | 564.667 | 240.306 | 103.690 |
| 2003 | RT | 1 | B | R | 1624.117 | 1269.461 | 989.603 | 597.724 | 429.883 | 291.536 | 59.320 | 16.355 | BQL |
| 2004 | RT | 1 | B | R | 1434.968 | 1201.942 | 977.590 | 535.062 | 441.081 | 252.590 | 101.694 | 26.023 | BQL |
| 2005 | RT | 1 | B | R | 2573.800 | 2194.998 | 1799.327 | 1107.928 | 868.609 | 482.488 | 197.145 | 51.323 | 18.829 |
| 2006 | TR | 2 | A | R | 3606.868 | 2938.697 | 2371.352 | 1875.820 | 1461.889 | 1137.446 | 663.812 | 262.510 | 108.426 |
| 2007 | RT | 1 | B | R | 2348.574 | 1955.589 | 1503.877 | 749.849 | 434.659 | 192.737 | 66.432 | 13.558 | BQL |
| 2008 | RT | 1 | B | R | 2699.000 | 2165.554 | 1936.232 | 1230.692 | 874.784 | 575.089 | 174.181 | 43.252 | BQL |
| 2009 | TR | 2 | A | R | 2704.673 | 2074.829 | 1490.527 | 879.451 | 670.284 | 480.435 | 243.011 | 94.491 | 39.511 |
| 2010 | RT | 1 | B | R | 1841.145 | 1459.459 | 1078.610 | 470.279 | 305.059 | 141.247 | 24.341 | BQL | BQL |
| 2011 | TR | 2 | A | R | 2021.377 | 1582.448 | 1287.058 | 671.930 | 357.538 | 150.272 | 48.426 | BQL | BQL |
| 2012 | TR | 2 | A | R | 1943.070 | 1762.876 | 1312.507 | 716.001 | 421.074 | 248.081 | 121.087 | 29.752 | 13.106 |
| 2013 | TR | 2 | A | R | 1700.099 | 1362.243 | 974.260 | 602.785 | 405.797 | 263.860 | 86.248 | 19.133 | BQL |
| 2014 | TR | 2 | A | R | 2572.230 | 2085.694 | 1602.485 | 981.360 | 653.165 | 465.139 | 174.794 | 55.311 | 21.839 |
| 2015 | RT | 1 | B | R | 3153.528 | 2768.628 | 2258.249 | 1477.317 | 1148.244 | 798.392 | 432.121 | 156.972 | 74.790 |
| 2016 | TR | 2 | A | R | 2903.044 | 2677.670 | 2408.555 | 1791.038 | 1564.826 | 1343.737 | 970.684 | 516.982 | 309.989 |
| 2017 | RT | 1 | B | R | 2912.959 | 2655.912 | 2329.727 | 1729.201 | 1581.025 | 1267.663 | 883.102 | 488.200 | 276.145 |
| 2018 | RT | 1 | B | R | 2636.744 | 2179.480 | 1722.155 | 983.123 | 754.296 | 467.669 | 179.368 | 61.336 | 27.899 |
| 2019 | RT | 1 | B | R | 2563.637 | 2129.867 | 1595.971 | 1056.044 | 739.138 | 445.288 | 139.846 | 26.467 | BQL |
| 2020 | TR | 2 | A | R | 1940.885 | 1570.957 | 1153.579 | 581.450 | 348.743 | 209.900 | 78.812 | 18.791 | 10.655 |
| 2021 | TR | 2 | A | R | 3102.689 | 2615.432 | 2210.600 | 1409.800 | 1139.477 | 989.104 | 590.378 | 296.482 | 153.437 |
| 2022 | RT | 1 | B | R | 3296.281 | 2782.425 | 2589.325 | 1843.680 | 1708.053 | 1199.268 | 598.644 | 218.449 | 73.541 |
| 2023 | TR | 2 | A | R | 2110.843 | 1883.451 | 1592.091 | 1137.052 | 1049.948 | 896.879 | 664.052 | 373.037 | 213.965 |
| 2024 | RT | 1 | B | R | 2500.057 | 2176.378 | 1822.998 | 1047.249 | 839.470 | 458.994 | 152.837 | 41.725 | 16.270 |
| n | | | | | 24 | 24 | 24 | 24 | 24 | 24 | 24 | 24 | 24 |
| Mean | | | | | 2487.9456 | 2099.4368 | 1703.1567 | 1082.8322 | 845.2989 | 592.3182 | 309.7032 | 129.6256 | 61.9479 |
| SD | | | | | 562.1876 | 510.7603 | 495.8146 | 437.9127 | 435.2869 | 382.8977 | 283.2356 | 155.6914 | 90.2679 |
| %CV | | | | | 22.60 | 24.33 | 29.11 | 40.44 | 51.50 | 64.64 | 91.45 | 120.11 | 145.72 |
| Min | | | | | 1434.968 | 1201.942 | 974.260 | 470.279 | 305.059 | 141.247 | 24.341 | 0.000 | 0.000 |
| Max | | | | | 3606.868 | 2938.697 | 2589.325 | 1875.820 | 1708.053 | 1343.737 | 970.684 | 516.982 | 309.989 |
